# Abdominal Pain Mimicking a Neurological Disorder: A Case Report of Spinal Cavernous Malformation in a Pediatric Patient

**DOI:** 10.7759/cureus.67525

**Published:** 2024-08-22

**Authors:** Eliézer Kasriel, Hans Boecher-Schwarz, Emmanuel Scalais

**Affiliations:** 1 Pediatric Neurology, Hôpital Civil Marie Curie, Charleroi, BEL; 2 Neurosurgery, Centre Hospitalier de Luxembourg, Luxembourg City, LUX; 3 Pediatric Neurology, Centre Hospitalier de Luxembourg, Luxembourg City, LUX

**Keywords:** pediatric, intramedullary lesion, cerebral cavernous malformation (ccm), spinal cord cavernous malformation, abdominal pain

## Abstract

We present a case of a 13-year-old boy with abdominal pain initially misdiagnosed as gastrointestinal in origin. Despite initial outpatient management, his symptoms rapidly deteriorated, revealing a central-medullary cavernous malformation causing spinal cord compression. This case underscores the importance of a comprehensive pediatric examination and highlights new treatment approaches for spinal cavernous malformations.

## Introduction

Abdominal pain is a common complaint in pediatric emergency departments, often attributed to gastrointestinal or musculoskeletal causes [1]. However, in rare cases, abdominal pain may be a manifestation of underlying neurological disorders, necessitating a thorough evaluation. We report a case of a 13-year-old boy with abdominal pain ultimately diagnosed with an intramedullary spinal cord cavernous malformation (ISCCM), emphasizing the importance of a comprehensive pediatric assessment.

## Case presentation

A 13-year-old boy, without any relevant personal or familial medical history, initially presented to the emergency department with left hypochondrial pain radiating to the back, following a dorsal impact during sports activity (soccer match) a week prior.

Over the subsequent week, the child consulted multiple times. He complained of crampy and epigastric pain, heartburn, and constipation. Clinical and complementary examinations (abdominal ultrasound, blood tests, stool, and urine analysis (refer to the Appendix for further details)) were unremarkable, and outpatient management with symptomatic treatment was every time pursued with no relief.

The child's symptoms deteriorated rapidly, with persistent pain that was unresponsive to over-the-counter analgesics. He complained of nocturnal awakenings due to abdominal pain, dysuria, dysesthesia, and weakness in the lower limbs. His gait became hesitant, even staggering.

On physical examination, nuchal stiffness was noted without signs of infection (no fever, no encephalopathy, etc.) or headaches, along with paravertebral and upper abdominal pain upon neck flexion. Additionally, we observed an ataxic gait, left lower limb steppage, instability on Romberg and monopodal stance, limited dorsiflexion of the feet, positive Mingazzini's test, and absence of cutaneous-abdominal reflexes. Sensory evaluation revealed paresthesia in the left lower limb and identified hypoesthesia extending up to the T5 dermatome, consistent with a sensory level.

**Figure 1:**
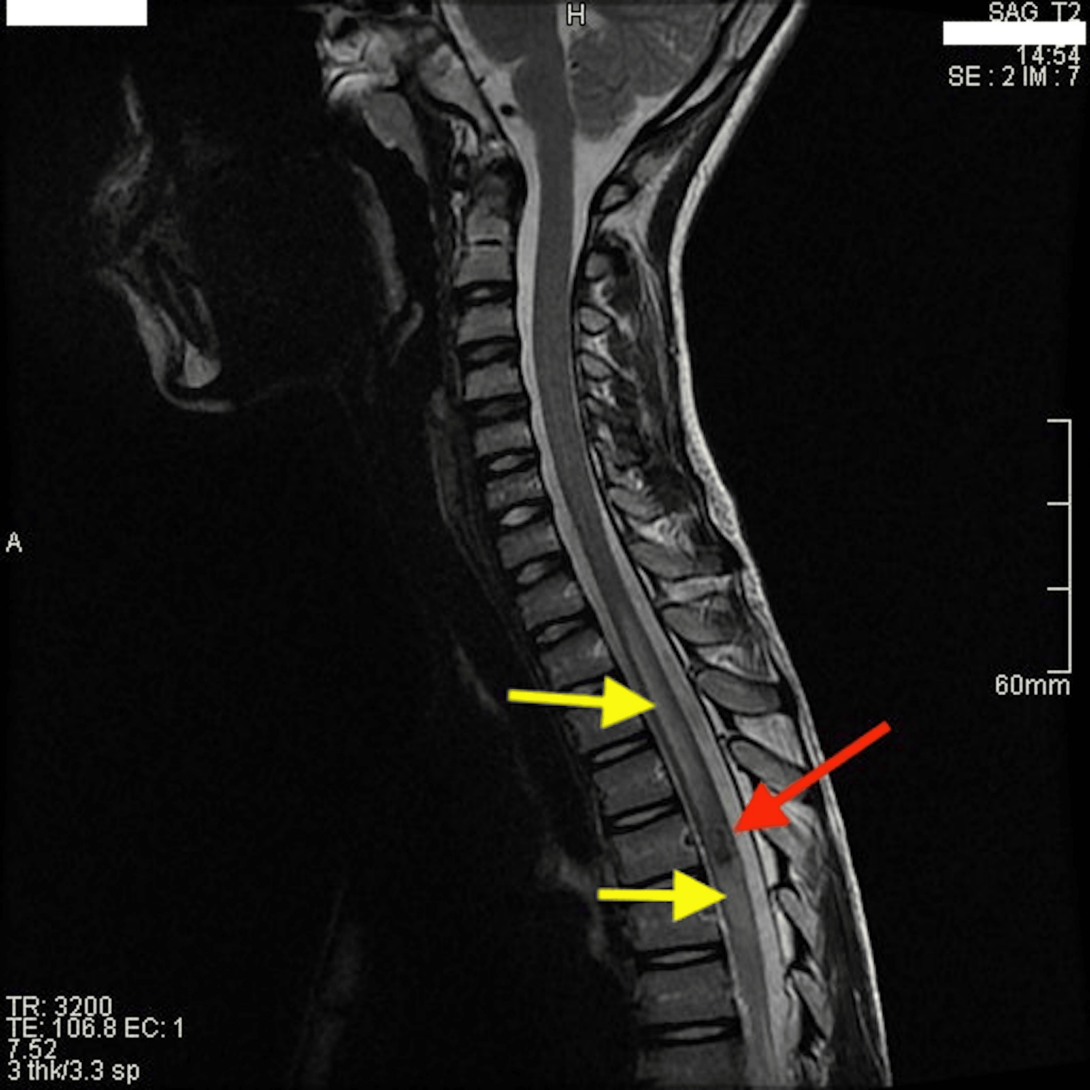
Intramedullary cavernous malformation on T2-weighted imaging. Sagittal T2-weighted MRI demonstrating a T5 cavernoma. Note the heterogeneous mixed signal intensity of the cavernous (red arrow) and the edematous perilesional reaction (yellow arrows).

**Figure 2:**
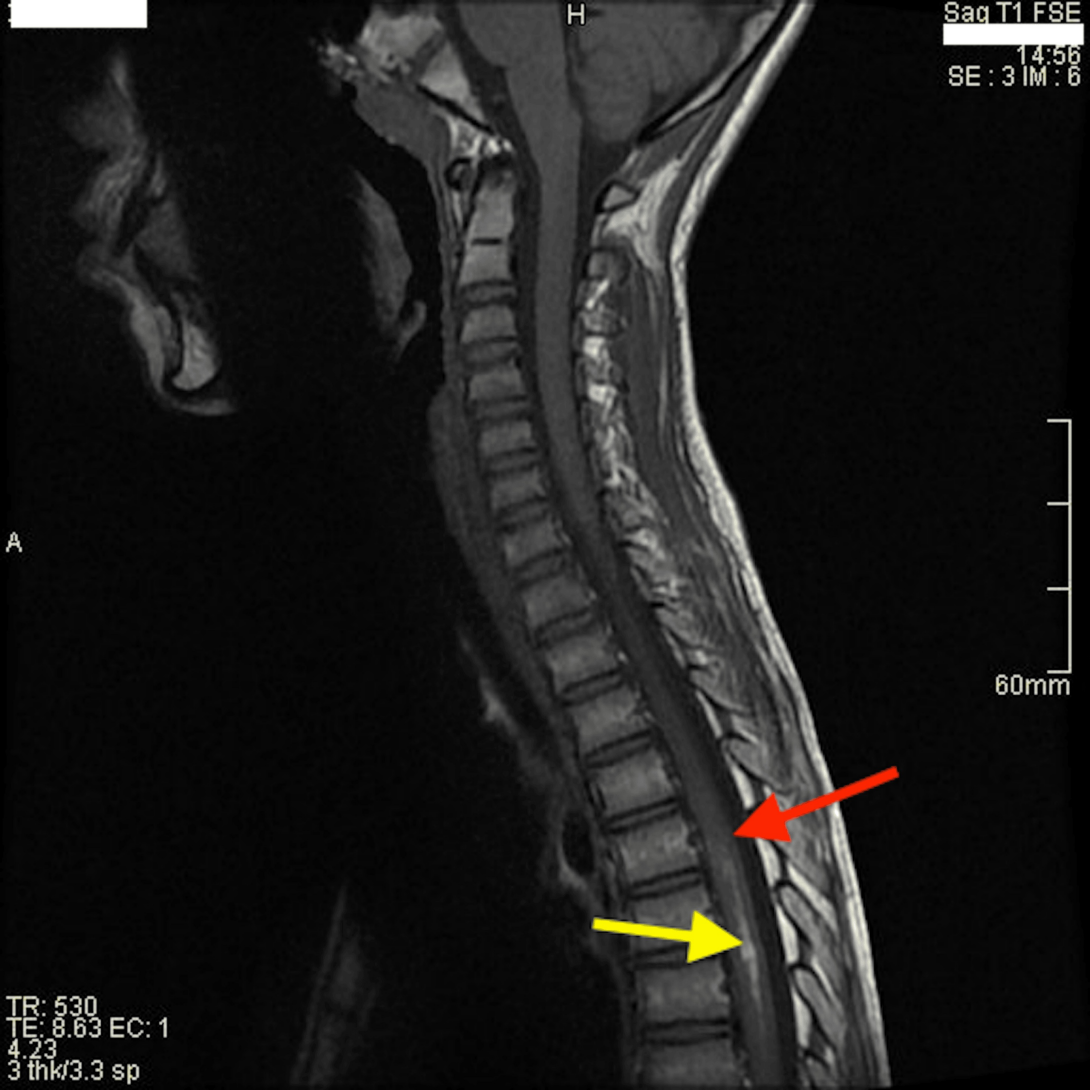
Underlying hemorrhage on T1-weighted imaging. Sagittal T1-weighted image with contrast product MRI demonstrating a non-enhancing isointense cavernoma (red arrow) and underlying hypersignal hemorrhage (yellow arrow).

Given a clinical picture suggestive of spinal cord compression syndrome, cerebral and spinal MRI was performed, revealing a central-medullary lesion at the level of the fifth thoracic vertebra. The lesion showed a heterogeneous hypointense signal on T2-weighted sequences associated with significant edematous reaction extending from T2 to T10 levels (Figure 1), markedly hypointense on T2 gradient echo, and isointense on T1-weighted sequences (Figure 2). There was underlying hemorrhage extending to T9 with hyperintensity on T1-weighted sequences.

The images suggested the presence of a centromedullary cavernous angioma measuring 11 x 5 mm.

Neurosurgical intervention was indicated following multidisciplinary consultation involving pediatric, neuroradiology, neurosurgery, and physical medicine and rehabilitation teams. The child's neurological status deteriorated shortly before surgical intervention, with a preoperative American Spinal Injury Association (ASIA) grade of C.

Excision of the intramedullary lesion was performed via T5 laminotomy under electrophysiological monitoring without complication. The child's neurological symptoms gradually improved, and he was discharged three days post surgery. Histopathological examination confirmed the diagnosis of cavernoma, revealing numerous dilated blood vessels with thin walls interlaced with fibrous tissue within gliotic tissue. Genetic analysis aimed at detecting pathogenetic variants associated with familial cavernoma formation yielded no positive findings. Postoperative follow-up was regular and multidisciplinary. Motor neurological recovery was complete after six months, and sphincter disorders resolved. The ASIA grade improved to A, although moderate abdominal pain, particularly during sports activity, and peri-umbilical paresthesia persisted.

**Figure 3:**
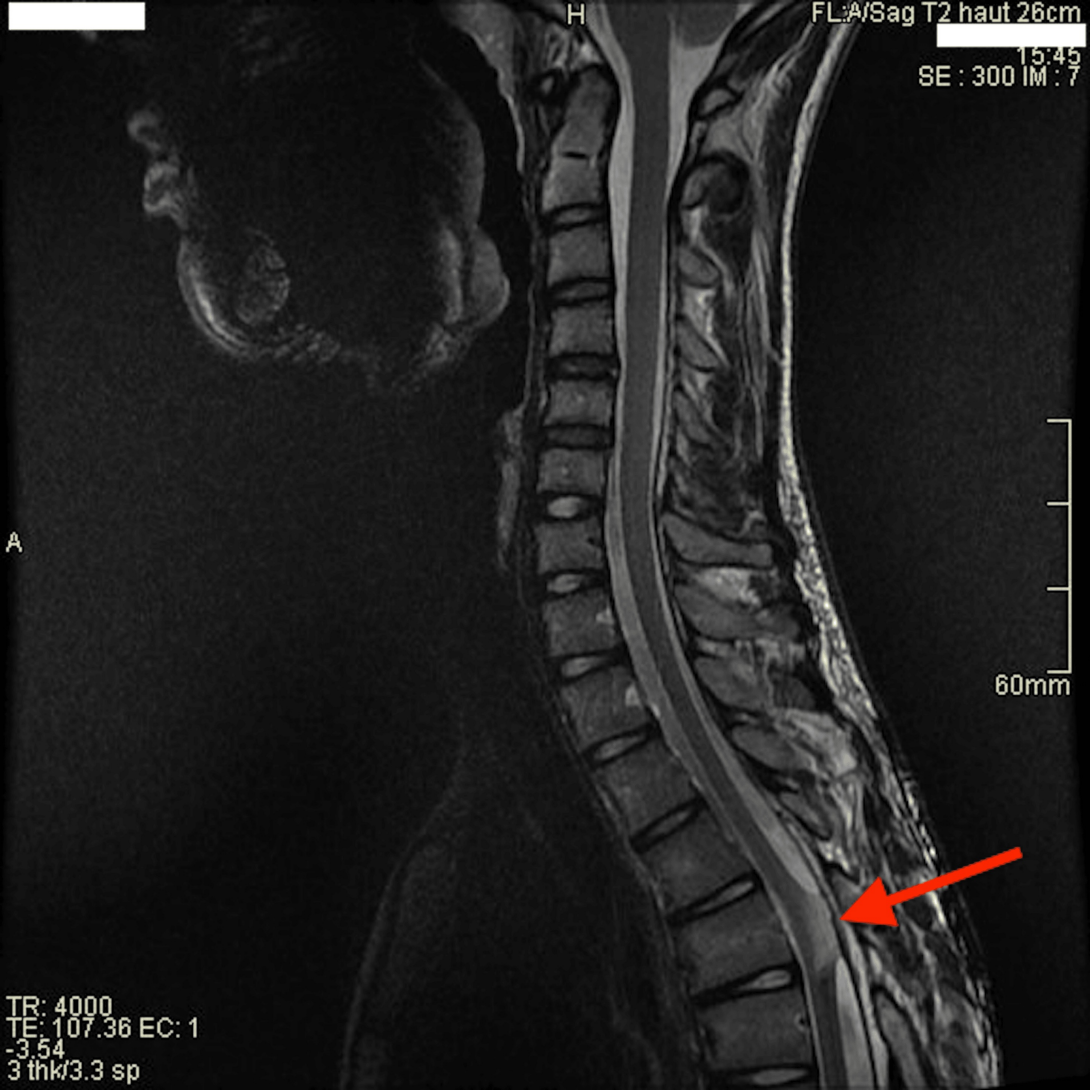
Sagittal T2-weighted image at five-year follow-up. After-effects of cavernous resection with retraction of the spinal cord backward (red arrow) at the laminotomy site.

After five years of follow-up, no recurrent hemorrhage was observed on various MRIs (Figure 3) performed for occasional abdominal pain, and the clinical examination remained entirely normal.

## Discussion

The presented case underscores the complexities involved in diagnosing abdominal pain in pediatric patients. Initial symptoms in our patient led to gastrointestinal-focused evaluation and delayed diagnosis of a rare but serious underlying condition.

Cavernomas are benign cerebrovascular malformations, commonly referred to as cavernous malformations, cryptic angiomas, or cavernous hemangiomas. They typically occur sporadically in 80% of cases, occasionally with a familial pattern in 20% [2]. Advances in genetic research have identified three mutated loci, due to loss of function mutation, predominantly in patients with multiple cerebral cavernomas or with a family history suggestive of this condition. The CCM1/KRIT1 gene, located on the long arm of chromosome 7, has primarily been described in Hispanic-American families. The CCM2/malcaverin gene, on the short arm of chromosome 7, and the CCM3/PDCD10 gene, located on 3q, have been found in non-Hispanic white families [3,4].

Macroscopically, the lesions, composed of tiny vessels clustered in a grape-like formation, resemble a blackberry, ranging in size from 2 mm to several centimeters in diameter. Microscopic examination reveals dilated vessels surrounded by endothelium and connective tissue. These vessels lack elastic fibers and smooth muscle. Generally, cerebral cavernomas do not consist of neural tissue [3].

The prevalence of cerebral cavernous malformations is not precisely known but is estimated to be between 0.1% and 0.5% in the general population. It is believed that these lesions account for 10% of cerebral vascular lesions and around 25% of all cavernomas are found in the pediatric population, without a gender predominance [5]. Symptoms depend on the lesion's location within the central nervous system and its size, often clinically manifesting after acute, subacute, or recurrent hemorrhage [5]. Due to the compact nature and high density of eloquent tissue, ISCCMs (1.5 cm) and brainstem cavernous malformations (2.7 cm) become symptomatic at smaller sizes compared to cavernomas in other locations [2].

ISCCMs rarely present asymptomatically (0.9%), with commonly reported manifestations including neuromotor deficits (60.6%), sensory deficits (57.8%), pain (33.8%), bowel or sphincter disturbances (23.6%), and respiratory distress (0.5%) [6,7]. They present with an acute neurological deficit (30%), stepwise (16%), or with a progressive neurological decline (54%) [6]. In children, symptoms often manifest as rapidly evolving neurological deficits following even minor trauma [5].

Several differential diagnoses should be considered, including multiple sclerosis, transverse myelitis, spinal neoplasms, or arteriovenous malformations, all of which can present with similar symptoms [6].

Currently, MRI is the diagnostic modality of choice. On T2-weighted imaging, the lesion appears heterogeneous with a "honeycomb" or “popcorn” pattern, sometimes surrounded by a hypointense halo, indicative of hemosiderin deposition, which is pathognomonic for the disease. The absence of enhancement also aids in radiological diagnosis [8].

Neurosurgical treatment with complete lesion excision is indicated for symptomatic or exophytic ISCCMs [6,9]. At long-term follow-up, postoperative outcomes are generally favorable, with neurological recovery observed in approximately 60% of cases, unchanged in 30%, and worse in 10% [6,7,9]. Functional walking is generally recovered two to three months after surgery [5].

In a systematic review, Badhiwala et al. demonstrated that the presence of sensory symptoms was associated with poor neurological recovery, whereas motor symptoms were correlated with better outcomes. They also found that for symptomatic ISCCMs, surgery within three months of symptom onset and gross-total resection were significant predictors of better neurological outcomes [7].

Ren et al. reported higher rates of hemorrhages and rehemorrhages in children with ISCCMs (8% and 30%/patient/year, respectively) compared to adults (2.8% and 7.4%, respectively). Rehemorrhagic events were described as significant predictors of an aggressive clinical presentation. They also found that thoracic or lumbar level lesions were associated with severe neurological and disability status [9].

In their five-year follow-up cohort of untreated ISCCMs, Santos et al. confirmed the increasing risk of hemorrhage and rebleeding of ISCCMs compared with their intracranial counterparts, potentially leading to worse neurological function [10].

Recent studies have explored alternative treatments for cavernous malformations. Propranolol, a beta-blocker, has shown promise in preclinical studies by reducing lesion development and improving barrier function [11]. Additionally, immune and inflammatory perspectives are being investigated to better understand the pathophysiology of cavernous malformations and identify potential targeted therapies [4]. Future research should focus on validating these new treatment approaches in pediatric populations and exploring their long-term efficacy and safety.

Half of pediatric patients with ISCCMs were initially misdiagnosed in Ren’s retrospective study, because of the broad initial symptoms and the rarity of ISCCMs, but also due to ignoring the importance of the neurological examination and neurological diagnosis, leading to failure of appropriate neuroimaging and treatment delays [9].

Our case emphasizes unique medullar semiology with sensory, motor, and sphincter disturbances leading to the discovery of a complex spinal lesion. Multidisciplinary collaboration ensured our patient received comprehensive care tailored to his individual needs, optimizing his quality of life and long-term outcomes. He demonstrated complete motor neurological recovery after six months and remained asymptomatic at the five-year follow-up, consistent with existing literature.

## Conclusions

This case illustrates the importance of a comprehensive pediatric examination in identifying rare neurological causes of abdominal pain. A comprehensive pediatric examination, including a thorough medical history, physical examination, and targeted diagnostic testing, is essential to ensure timely diagnosis and appropriate management.

## Appendices

**Table 1:** First blood test in the pediatric emergency unit after three days of abdominal pain.

Blood biomarkers	Value	Units of measurement	Normal reference values
White blood cells	13.33	Number x 10^9/L	4.50-11.40
Red blood cells	4.58	Number x 10^12/L	4.10-5.55
Hemoglobin	12.5	g/dL	12.5-16.0
Hematocrit	37.5	%	36.5-47.5
Mean corpuscular volume	81.9	fL	78.0-93.0
Mean corpuscular hemoglobin	27.3	pg	26.0-32.5
Mean corpuscular hemoglobin concentration	33.3	g/dL	31.5-36.0
Platelets	262	Number x 10^9/L	170-400
Mean platelet volume	8.8	fL	9.3-12.1
Neutrophils	10.42	Number x 10^9/L	1.70-7.90
Lymphocytes	1.46	Number x 10^9/L	1.20-5.00
Monocytes	1.30	Number x 10^9/L	0.10-0.95
Eosinophils	0.06	Number x 10^9/L	0.02-0.65
Basophils	0.05	Number x 10^9/L	<0.20
Granulocytes	0.04	Number x 10^9/L	<0.10
Urea	22	mg/dL	18-45
Creatinine	0.64	mg/dL	0.46-0.81
Sodium	138	mmol/L	136-145
Potassium	4.4	mmol/L	3.5-5.1
Chlorine	99	mmol/L	98-107
Calcium	10.0	mmol/L	8.4-10.2
Inorganic phosphate	4.5	mmol/L	2.9-5.1
C-reactive protein	27.9	mg/L	<5.0
Glutamic oxaloacetic transaminase	21	U/L	<38
Glutamic pyruvic transaminase	11	U/L	<41
Gamma-glutamyl transferase	12	U/L	<43
Lactate dehydrogenase	233	U/L	<279
Lipase	12	U/L	13-60
Creatine phosphokinase	67	U/L	<270
Alkaline phosphatase	170	U/L	116-468

**Table 2:** Second blood test the following day after four days of abdominal pain.

Blood biomarkers	Value	Units of measurement	Normal reference values
White blood cells	13.33	Number x 10^9/L	4.50-11.40
Red blood cells	4.58	Number x 10^12/L	4.10-5.55
Hemoglobin	12.5	g/dL	12.5-16.0
Hematocrit	37.5	%	36.5-47.5
Mean corpuscular volume	81.9	fL	78.0-93.0
Mean corpuscular hemoglobin	27.3	pg	26.0-32.5
Mean corpuscular hemoglobin concentration	33.3	g/dL	31.5-36.0
Platelets	262	Number x 10^9/L	170-400
Mean platelet volume	8.8	fL	9.3-12.1
Neutrophils	10.42	Number x 10^9/L	1.70-7.90
Lymphocytes	1.46	Number x 10^9/L	1.20-5.00
Monocytes	1.30	Number x 10^9/L	0.10-0.95
Eosinophils	0.06	Number x 10^9/L	0.02-0.65
Basophils	0.05	Number x 10^9/L	<0.20
Granulocytes	0.04	Number x 10^9/L	<0.10
Urea	22	mg/dL	18-45
Creatinine	0.64	mg/dL	0.46-0.81
Sodium	138	mmol/L	136-145
Potassium	4.4	mmol/L	3.5-5.1
Chlorine	99	mmol/L	98-107
Calcium	10.0	mmol/L	8.4-10.2
Inorganic phosphate	4.5	mmol/L	2.9-5.1
C-reactive protein	27.9	mg/L	<5.0
Glutamic oxaloacetic transaminase	21	U/L	<38
Glutamic pyruvic transaminase	11	U/L	<41
Gamma-glutamyl transferase	12	U/L	<43
Lactate dehydrogenase	233	U/L	<279
Lipase	12	U/L	13-60
Creatine phosphokinase	67	U/L	<270
Alkaline phosphatase	170	U/L	116-468

**Table 3:** Blood test before neurosurgery.

Blood biomarkers	Value	Units of measurement	Normal reference values
White blood cells	11.53	Number x 10^9/L	4.50-11.40
Red blood cells	4.80	Number x 10^12/L	4.10-5.55
Hemoglobin	12.80	g/dL	12.5-16.0
Hematocrit	38.5	%	36.5-47.5
Mean corpuscular volume	80.2	fL	78.0-93.0
Mean corpuscular hemoglobin	26.7	pg	26.0-32.5
Mean corpuscular hemoglobin concentration	33.2	g/dL	31.5-36.0
Platelets	443	Number x 10^9/L	170-400
Mean platelet volume	9.0	fL	9.3-12.1
Neutrophils	7.99	Number x 10^9/L	1.70-7.90
Lymphocytes	1.79	Number x 10^9/L	1.20-5.00
Monocytes	1.57	Number x 10^9/L	0.10-0.95
Eosinophils	0.00	Number x 10^9/L	0.02-0.65
Basophils	0.03	Number x 10^9/L	<0.20
Granulocytes	0.15	Number x 10^9/L	<0.10
Sodium	135	mmol/L	136-145
Potassium	4.4	mmol/L	3.5-5.1
International normalized ratio	1.00	N/A	<1.30
Activated partial thromboplastin time	0.87	seconds	0.75-1.20
Fibrinogen	2.27	g/L	2.00-4.00
Blood type	A Rhesus(D) +, Ccee, Kell -	N/A	N/A

**Table 4:** Blood test after neurosurgery.

Blood biomarkers	Value	Units of measurement	Normal reference values
White blood cells	15.82	Number x 10^9/L	4.50-11.40
Red blood cells	4.11	Number x 10^12/L	4.10-5.55
Hemoglobin	11.2	g/dL	12.5-16.0
Hematocrit	33.4	%	36.5-47.5
Mean corpuscular volume	81.3	fL	78.0-93.0
Mean corpuscular hemoglobin	27.3	pg	26.0-32.5
Mean corpuscular hemoglobin concentration	33.5	g/dL	31.5-36.0
Platelets	333	Number x 10^9/L	170-400
Mean platelet volume	8.9	fL	9.3-12.1
Neutrophils	11.84	Number x 10^9/L	1.70-7.90
Lymphocytes	1.86	Number x 10^9/L	1.20-5.00
Monocytes	1.95	Number x 10^9/L	0.10-0.95
Eosinophils	0.00	Number x 10^9/L	0.02-0.65
Basophils	0.02	Number x 10^9/L	<0.20
Granulocytes	0.15	Number x 10^9/L	<0.10

**Table 5:** CSF analysis before surgery.

CSF and blood biomarkers	Value	Units of measurement	Normal reference values
White blood cells in CSF	3	/µL	<10
Red blood cells in CSF	<1	/µL	<1
Glucose in CSF	77	mg/dL	45-70
Proteins in CSF	0.23	g/L	<0.50
Albumin in CSF	14.2	mg/dL	<40.0
IgG in CSF	1.6	mg/dL	<3.5
IgA in CSF	1.0	mg/L	<5.0
IgM in CSF	0.26	mg/L	<1.30
Isofocusing	None	N/A	N/A
Lactate in CSF	16.7	mg/L	<25.2
Bacterial detection in CSF	Negative	N/A	N/A
Serum IgG	9.29	g/L	5.60-15.70
Serum IgA	1.14	g/L	0.86-5.44
Serum IgM	0.88	g/L	0.33-1.35
Serum albumin	49	g/L	37-53

**Table 6:** Stool analysis in the pediatric emergency unit.

Stool Biomarkers	Value	Units of measurement	Normal reference values
Bacterial detection	Negative	N/A	N/A
H. pylori antigen	Negative	N/A	N/A

**Table 7:** Urine analysis in the pediatric emergency unit.

Urine biomarkers	Value	Units of measurement	Normal reference values
pH	7.0		5.5-7.5
White blood cells	<1	/µL	<12
Red blood cells	<1	/µL	<5
Nitrite	Negative	N/A	N/A
Proteins	Negative	N/A	N/A
Ketone	Negative	N/A	N/A
Bacterial detection	Negative	N/A	N/A

**Table 8:** Blood culture in the pediatric emergency unit.

Blood culture	Value	Units of measurement	Normal reference values
Bacterial detection	Negative	N/A	N/A
